# The risk of new-onset diabetes in antidepressant users – A systematic review and meta-analysis

**DOI:** 10.1371/journal.pone.0182088

**Published:** 2017-07-31

**Authors:** Virginio Salvi, Ilaria Grua, Giancarlo Cerveri, Claudio Mencacci, Francesco Barone-Adesi

**Affiliations:** 1 Department of Neuroscience, ASST Fatebenefratelli-Sacco, Milan, Italy; 2 Department of Pharmaceutical Sciences, University of Eastern Piedmont, Novara, Italy; McMaster University, CANADA

## Abstract

**Background:**

Antidepressant Drugs (ADs) are among the most commonly prescribed medications in developed countries. The available epidemiological evidence suggests an association between AD use and higher risk of developing type 2 diabetes mellitus. However, some methodological issues make the interpretation of these results difficult. Moreover, very recent studies provided conflicting results. Given the high prevalence of both diabetes and AD use in many countries, clarifying whether this association is causal is of extreme relevance for the public health. The aim of the present study is to provide an up-to-date evaluation of the evidence in support of a causal role of ADs in inducing diabetes.

**Methods and findings:**

A systematic literature search was conducted to identify relevant studies in MEDLINE (PubMed), PsycINFO, and International Pharmaceutical Abstracts (IPA) through 31^st^ December 2016. Only studies assessing the incidence of new-onset diabetes in subjects treated with ADs were included. Results were pooled using a random-effects meta-analysis. Moreover, we extensively reviewed the role of the different sources of bias that have been proposed to explain the association between AD and diabetes. Twenty studies met the inclusion criteria. In the meta-analysis, the association between AD use and diabetes was still evident after the inclusion of the recent negative studies [pooled relative risk = 1.27, 95% confidence interval (CI), 1.19–1.35; p<0.001]. None of the biases proposed by previous authors seemed able to fully explain the observed association.

**Conclusions:**

This updated meta-analysis confirms the association between AD use and incident diabetes. It still remains a matter of debate whether single ADs exert a different effect on the risk of diabetes. Given the possible heterogeneity, we suggest that a classification of ADs according to their pharmacological profiles could be useful in better elucidating the nature of this association.

## Introduction

Antidepressant drugs (ADs) use strongly increased over the last 20 years in developed countries, with around 13% of the US population currently prescribed ADs [1, 2]. This issue is becoming of utmost importance also in developing countries, where depression is one of the leading causes of disability [3]. In recent years, mounting evidence has linked AD use with type 2 diabetes mellitus [4, 5]. Different mechanisms are called upon to explain this association: the use of some ADs is associated with weight gain [6], a well-known risk factor for diabetes. Moreover, other pathophysiological mechanisms directly linking ADs and hyperglycemia have been postulated, especially for adrenergic ADs as the tricyclics [5].

Two previous meta-analyses reported a 1.5-fold increase in risk of diabetes in patients treated with ADs compared to the general population [7, 8]. However in recent years new studies have been published, some of which failed to confirm this association [9–11]. Furthermore, recent highly phenotyped longitudinal studies failed to find an association between exposure to ADs and modifications in glucose levels over time. This prompted some authors to suggest that the association between ADs and diabetes could be in fact spurious, due to the fact that patients receiving AD treatment may be more likely to have a blood glucose test, thereby increasing the chance of a diabetes diagnosis [12–14]. Moreover, the fact that depression itself can increase the risk of diabetes and hence confound the association between ADs and diabetes further complicates the issue [15].

Given the very high prevalence of AD use and diabetes in the general population, clarifying this matter is of extreme relevance for public health. Aim of the present study is therefore to update the previous meta-analyses and to provide a comprehensive evaluation of the risk of diabetes associated to AD use.

## Methods

### Search strategy

A systematic literature search was conducted in PubMed, PsycINFO, and International Pharmacological Abstracts (IPA) on 10^th^ June 2017 to identify eligible studies published between 2000 and 2016 reporting associations between AD use and diabetes onset. The search strings showing the search strategy are reported in S1 Appendix. In addition, we searched the references of all articles included in the meta-analysis to identify additional studies of interest.

A protocol was not registered and ethics review was not required for conducting this study.

### Inclusion and exclusion criteria

The references were selected by hand according to the following inclusion criteria:

1. Studies assessing the risk of new-onset diabetes among AD users compared with non-users. Thus, cross-sectional studies were excluded.
2. Studies on subjects aged at least 18 years
3. Studies published in English language only
4. Studies published between 1^st^ January 2000 and 31^st^ December 2016

Study selection and data extraction were performed by the first two authors (VS and IG). Disagreements were resolved by reaching a consensus through discussion. In case consensus could not be reached, the last author (FBA) acted as an arbitrator.

### Data extraction

The following data were extracted from the included studies: name of first author, year of publication, country, study design, study period, type of ADs used (if specified), covariates used for adjustment or stratification. Selection of the estimates from each study was conducted using a strategy previously adopted in other systematic reviews [16]. Only one effect estimate per study was included in the meta-analysis. When more than one estimate was available, the one adjusted for the largest number of possible confounders was preferred. When only results from subgroup analyses (for example according to short-term/long-term use of AD or severity of depressive symptoms) were available in a study, we calculated a pooled estimate for all groups combined and included it in our meta-analysis. Nevertheless, results from subgroup analyses were recorded anyway and used, when relevant, in secondary analyses. When estimates from different papers were based on data from the same study, the estimates based on the larger number of cases (usually the most recent one) were chosen.

### Assessment of study quality

The methodological quality of included studies was assessed independently by the first and last author (VS and FBA) using the Newcastle-Ottawa Scale (NOS) for quality of case control and cohort studies in meta-analyses. All disagreements were resolved by discussion until a consensus was reached. The NOS comprises nine items grouped in three subscales: selection of study groups, comparability of groups, and ascertainment of outcome (cohort studies) or exposure (case-control studies) [17]. A score system based on “stars” (0–9 stars) has been developed for assessment. The subscale “comparability of groups” gives two stars when the study controls for two confounders considered to be relevant for the studied association. Since both AD use and diabetes are more frequent in overweight and obese patients, we decided to give the first star to studies controlling for baseline BMI. Moreover, since depression itself, which constitutes the most important indication for AD use, is an acknowledged risk factor for diabetes, we gave the second star in the “comparability” subscale to studies controlling for presence of depression or, when available, depressive severity assessed by rating scales. In the current study, we considered a study awarded a star score ≥ 8 as a high quality study.

### Statistical analysis

We calculated summary effects estimates using fixed and random effects models [18], and heterogeneity using the I^2^ statistic [18]. The I^2^ statistic was categorized as either small (from 25% to <50%), medium (from 50% to <75%) or large (≥75%) [19]. Publication bias was evaluated examining the funnel plots [20] and through two formal tests [21, 22]. We carried out secondary analyses to evaluate the robustness of our estimates. We conducted the analysis stratifying by type of AD used (SSRI vs non-SSRI), country where the study was conducted (USA vs other countries), study design (cohort/RCT vs nested case-control), source of information about AD treatment (self-report vs medical prescriptions), source of information about the diagnosis of diabetes (self-report vs antidiabetics prescriptions or clinical diagnosis) and NOS score (less than 8 vs 8 or more). Moreover we replicated the analysis including only studies adjusting for BMI and presence of depression or, when available, severity of depressive symptoms. Finally, a sensitivity analysis was conducted using the leave-one-out approach.

## Results

### Study characteristics

**Fig 1:**
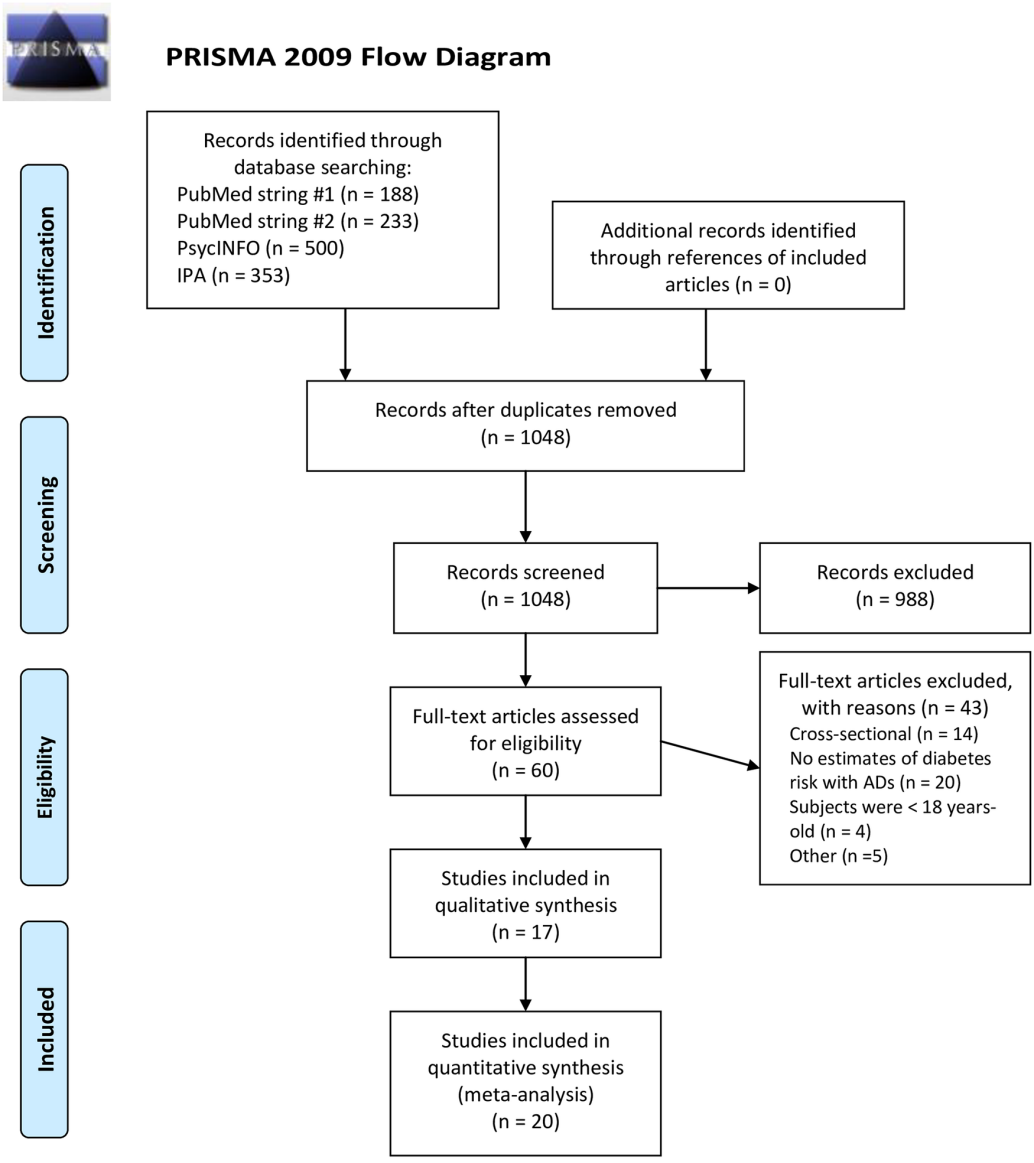
PRISMA Flow-Diagram of the systematic review. *Form*: Moher D, Liberati A, Tetzlaff J, Altman DG, The PRISMA Group (2009). *P*referred *R*eporting *i*tems for *S*ystematic Reviews and *Meta*-*A*nalyses: The PRISMA Statement. PLoS Med 6(7): e1000097. doi:10.1371/journal.pmed1000097. **For more information, visit** www.prisma-statement.org.

The primary electronic literature search identified 1274 articles from PubMed, PsycINFO, and IPA (Fig 1). After duplicates removal, 1048 articles were considered for screening. Out of these, screened according to title and abstract, only 60 articles were eligible for full-text analysis. Of these, 43 articles were excluded because they didn’t match the inclusion criteria. Thus, 17 papers met our selection criteria. Two papers reported results from more than one study [23, 24]; the reported estimates of the single studies used by Pan and Frisard were used in the present analysis. Thus, 20 estimates were included in the main analysis (Table 1). Five studies previously included in the meta-analyses of Yoon and Bhattacharjee were excluded from the present review [7, 8]. For two of these studies updated results were available [10, 24], and thus they were included in our meta-analysis instead of the old ones [25, 26]. The remaining three studies were excluded because one was restricted to children and adolescents [27], one had a cross-sectional design [28] and the last one was based on spontaneous reports of hyperglycemia rather than on new-onset diabetes [29]. Among the 20 studies included in our systematic review, fourteen used a cohort design [9–12, 23, 24, 30–35], four used a nested case–control study design [4, 36–38], two were randomized controlled trials (RCT) [24, 39]. Eleven studies were from North America (USA and Canada), six from Europe, two from Asia and one from Australia. The methods of exposure assessment to ADs were: patients bringing AD packages at medical visits (2 studies) [24, 39], review of clinical charts (one study) [35], electronic records of drug prescriptions (7 studies) [4, 11, 33–37], structured interviews (4 studies) [10, 12, 31, 32], and self-report (6 studies) [9, 23–25]. Diabetes status was assessed through independent clinical diagnosis (4 studies) [12, 35, 38, 39], record linkage (6 studies) [4, 11, 33, 34, 36, 37], and self-report (10 studies) [9, 10, 23, 24, 31, 32, 35]. The mean value for the methodological quality of the included 20 studies using the NOS was 7.05 stars. Only one study had less than 6 stars. The most common NOS item lacking a star was “Assessment of Outcome”.

**Table 1:** Characteristics of studies included in the meta-analysis.

Study and year	Country	Study design	Duration of follow-up (years)	Diabetes cases	RR (95% CI)	Adjustment variables	Quality of studies
Andersohn 2009 [4]	UK	Nested case-control	15	2243	1.40 (1.16–1.70)	BMI, smoking, hypertension, hyperlipidemia, recent use of beta-blockers, thiazides, antipsychotics, carbamazepine, phenytoin, valproate, lithium, glucocorticoids	9
Atlantis 2010 [30]	Australia	Cohort	10	155	1.80 (0.91–3.57)	Demographic and lifestyle factors, functional health, and prevalent chronic disease	8
Bhattacharya 2014 [10]	USA	Cohort	1	525	1.06 (0.77–1.47)	Age, gender, race/ethnicity, presence of depression, lifestyle risk factors—BMI, physical activity, smoking status, poverty status, insurance status	6
Campayo 2010 [31]	Spain	Cohort	5	163	1.26 (0.63–2.50)	Diabetes risk factors and AD and antipsychotic use	8
Chang 2015 [11]	Korea	Cohort	4	426	0.75 (0.50–1.12)	Age, gender, education, Charlson comobidity index, BMI, mini-mental state examination (MMSE), geriatric depression scale (GDS)	7
Frisard 2015 (WHI-CT) [24]	USA	RCT	8	4171	1.27 (1.13–1.43)	Age, ethnicity, education, physical activity, total energy intake, propensity for AD medication use, hormone replacement therapy, elevated depressive symptoms, BMI	8
Frisard 2015 (WHI-OS) [24]	USA	Cohort	8	3624	1.35 (1.21–1.51)	Age, ethnicity, education, physical activity, total energy intake, propensity for AD medication use, hormone replacement therapy, elevated depressive symptoms, BMI	7
Khoza 2012 [32]	USA	Cohort	7	2937	1.56 (1.40–1.73)	Age, gender, medication adherence, number of concomitant diabetogenic medications, more recent year of cohort entry	6
Kisely 2009 [36]	Canada	Nested case-control	5	608	1.12 (0.90–1.40)	Age, gender, previous health service use	7
Kivimäki 2010 [37]	Finland	Nested case-control	4	851	1.77 (1.37–2.30)	Prevalent physical disease (hypertension, coronary heart disease, cerebrovascular disease, and cancer)	8
Kivimäki 2011 [12]	UK	Cohort	18	346	1.24 (0.54–2.87)	Age, gender, and ethnicity	9
Knol 2007 [33]	The Netherlands	Cohort	7	499	1.06 (0.89–1.26)	Age, gender, Chronic Disease Score (heart disease, respiratory illness, cancer, ulcer, high cholesterol)	7
Pan 2012 (HPFS) [23]	USA	Cohort	16	1287	1.37 (1.07–1.76)	Age, ethnicity, marital status, living status, smoking, alcohol intake, multivitamin and aspirin use, physical activity, family history of diabetes, major comorbidities, dietary score, BMI	5
Pan 2012 (NHS I) [23]	USA	Cohort	12	3514	1.08 (0.97–1.19)	Age, ethnicity, marital status, living status, smoking, alcohol intake, multivitamin and aspirin use, physical activity, family history of diabetes, major comorbidities, dietary score, BMI, MHI-5 score	6
Pan 2012 (NHS II) [23]	USA	Cohort	14	1840	1.21 (1.08–1.35)	Age, ethnicity, marital status, living status, smoking, alcohol intake, multivitamin and aspirin use, physical activity, family history of diabetes, major comorbidities, dietary score, BMI, MHI-5 score	6
Pérez-Piñar 2016 [34]	UK	Cohort	10	4223	1.32 (1.29–1.34)	Age, gender, ethnicity, psychiatric diagnosis, antipsychotics, Townsend score for social deprivation	7
Rubin 2010 [39]	USA	RCT	10	N/A	2.41 (1.63–3.57)	Age, gender, race/ethnicity, education, fasting plasma glucose at baseline, weight at baseline, and weight change	7
Sambamoorthi 2013 [9]	USA	Cohort	4	467	0.91 (0.66–1.26)	Gender, race/ethnicity, education, poverty status, prescription drug insurance, health status, functional status, BMI, smoking, presence of heart disease and hypertension	7
Vimalananda 2014 [35]	USA	Cohort	12	3372	1.26 (1.11–1.43)	Age, questionnaire cycle, healthcare utilization, family history of diabetes, years of education, lifestyle factors (vigorous activity levels, daily hours of television watching, caloric intake, smoking, and alcohol consumption, BMI	6
Wu 2014 [38]	Taiwan	Nested case-control	12	47885	1.20 (1.05–1.37)	Age, gender, comorbidity with hypertension or hyperlipidemia, presence of mood disorders, use of antipsychotics	7

### Risk of DM by using ADs

**Fig 2:**
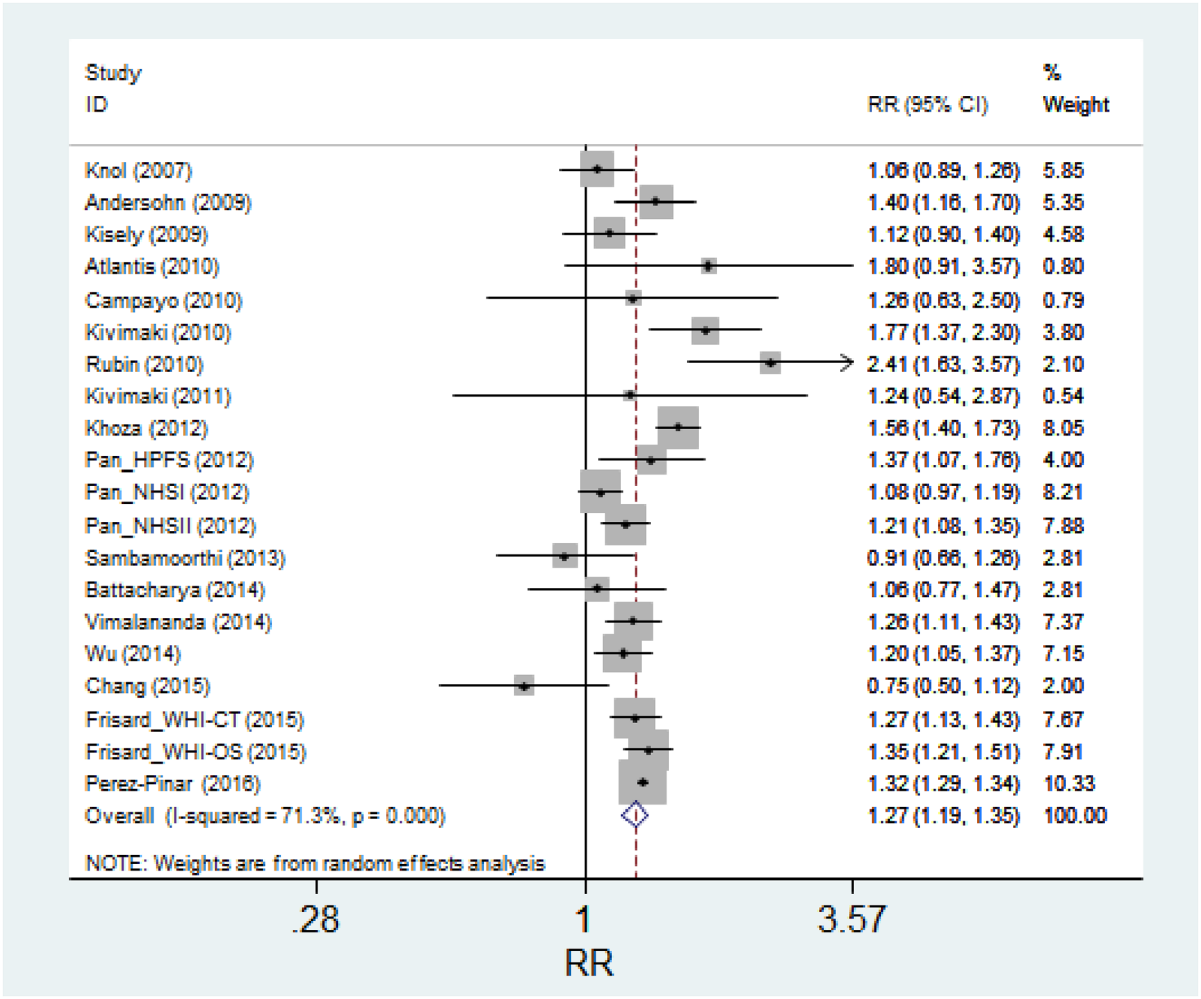
Random effects meta-analysis of the association between use of ADs and incidence of diabetes.

All the studies but two [9, 11] showed an association between AD use and diabetes, albeit this reached the nominal statistical significance only in 11 studies. In a random effects meta-analysis, a statistically significant association between AD use and diabetes was observed (Pooled Relative Risk: 1.27, 95% CI: 1.19 to 1.35; p<0.001) (Fig 2). There was substantial heterogeneity among the studies (I^2^ = 71%). In all the analyses funnel plots were symmetrical and neither the Begg’s nor Egger’s tests suggested publication bias (p>0.20 for both tests). Several secondary analyses were conducted to evaluate the robustness of the main analysis, but results did not change appreciably (Table 2). In particular results did not differ among studies using different methods of exposure or outcome assessment. We also did not find evidence of heterogeneity between cohort studies and case-control studies. When we restricted the analyses to the six high-quality studies, defined by a high NOS score of 8 or 9, relative risk further increased to 1.40. Also, results did not change substantially when we conducted the analysis including only studies controlling for BMI and presence of depression/severity of depressive symptoms. Finally, a sensitivity analysis based on the leave-one-out method did not change the results appreciably. Results remained statistically significant after the exclusion of each study and the point estimates changed minimally, ranging between 1.25 and 1.28.

**Table 2:** Overall and subgroup analyses of the association between use of ADs and incidence of diabetes.

Stratification	Groups	No. of studies	Fixed effect model	Random effect model	I^2^ (%)
Overall studies		20	1.31 (1.28–1.33)	1.27 (1.19–1.35)	71
Type of antidepressant					
	SSRI	7	1.23 (1.15–1.31)	1.21 (1.07–1.37)	70
	Non-SSRI	7	1.40 (1.32–1.48)	1.31 (1.16–1.47)	65
Country					
	USA	10	1.28 (1.23–1.34)	1.28 (1.16–1.43)	78
	Non-USA	10	1.31 (1.29–1.34)	1.25 (1.13–1.39)	62
Type of study					
	Cohort	16	1.31 (1.28–1.33)	1.26 (1.17–1.35)	74
	Nested Case Control	4	1.29 (1.18–1.42)	1.33 (1.12–1.58)	67
Source of information about AD treatment					
	Self-report	12	1.23 (1.17–1.29)	1.25 (1.14–1.37)	59
	Prescriptions	8	1.32 (1.29–1.34)	1.28 (1.16–1.43)	78
Source of information about the diagnosis of diabetes					
	Self-report	9	1.23 (1.17–1.29)	1.23 (1.15–1.32)	34
	Antidiabetics prescriptions or clinical diagnosis	11	1.32 (1.29–1.34)	1.29 (1.16–1.44)	79
Study Quality	NOS <8	14	1.30 (1.28–1.33)	1.24 (1.15–1.33)	78
	NOS 8–9	6	1.36 (1.24–1.49)	1.40 (1.24–1.57)	19
Adjustment for specific risk factors					
	BMI	11	1.22 (1.16–1.28)	1.21 (1.12–1.31)	54
	Depression	13	1.30 (1.28–1.33)	1.25 (1.16–1.34)	68
	BMI and depression	10	1.21 (1.16–1.27)	1.20 (1.10–1.30)	57

## Discussion

In our meta-analysis we found an association between exposure to ADs and new-onset diabetes, with a relative risk of 1.27. When we restricted the analysis to the studies to high NOS score the association between ADs and diabetes was even stronger (Table 2).

The results are in line with those from two previous meta-analyses that reported a 1.5-fold increase of diabetes among AD users [7, 8]. These meta-analyses retrieved data from studies up to 2012, and since then five cohort studies [9–11, 34, 35] and one nested case-control study [38] have been conducted. Of these, three did not find any association between AD use and diabetes [9–11]. Although attenuated compared to previous results, our study still confirms the association between ADs and incident diabetes, again suggesting caution when prescribing these agents to patients at risk for diabetes mellitus.

In past years, most research on the metabolic effects of psychotropics was focused on antipsychotics, especially second-generation ones, with their well-acknowledged ability to induce metabolic syndrome and diabetes [40–43]. On the contrary, ADs were considered to be neutral or even beneficial on glucose homeostasis, having previous studies demonstrated that AD use leads to improvements in glucose and insulin levels over the short-term [44, 45]. However, some authors suggested that improvement in insulin sensitivity was due to resolution of depressive symptoms and not merely to AD exposure, since only responders and remitters to ADs showed it [45]. On the other hand, in a recently published study better insulin sensitivity was only associated with SSRIs whereas use of tricyclics was associated with higher HOMA-IR scores [46], likely supporting previous hypotheses by which ADs with adrenergic and cholinergic activity may determine an increase in glucose [5, 47].

The putative mechanism by which some ADs may worsen glucose metabolism could involve weigh gain, which could occur even in the short-term. In a meta-analysis conducted on 116 studies, the authors found out that some, although not all, ADs could significantly increase weight even within the first 12 weeks of treatment, further increasing body weight over the long-term [6]. Since weight gain is probably the most relevant determinant of diabetes through the induction of insulin-resistance, the observed association between AD use and diabetes is not surprising.

On the other hand, the observed association between ADs and diabetes may have several alternative explanations. First, diabetes itself can trigger the risk for depression and lead, by reverse causation, to the prescription of ADs. However, this problem is relevant mainly in cross-sectional studies, where the direction of causality cannot be firmly established. For this reason we included in our meta-analysis only prospective studies evaluating the incidence of new-onset diabetes over time.

Secondly, results could be due to confounding: ADs are typically prescribed to patients who often engage in unhealthy lifestyles such as unbalanced diet, either poor or, in case of atypical depression, characterized by overeating of high glycemic index carbohydrates. Asthenia and lack of motivation also lead to a marked decrease of physical activity in these patients. Furthermore, these people often fail to attend medical examinations and checkups, thereby increasing the likelihood of developing lipid metabolism disturbances and diabetes. Finally, depression itself can directly increase diabetes risk, for instance by triggering the disruption in the regulation of pro-inflammatory cytokines, such as TNF-alpha, whose presence is high in patients with diabetes [48, 49]. In an attempt to control for the confounding effect of depression, we carried out secondary analyses including only studies that adjusted for both BMI and the presence/severity of depression: results did not change appreciably, suggesting that confounding could play a lesser role in this case (Table 2). However, it has to be noted that residual confounding cannot be completely ruled out. Indeed, residual symptoms of depression such as fatigue and sedation may be relevant in patients with low depression scores [50], even during remission [51]. Residual symptoms such as fatigue and lack of energy, which easily turn into reduced physical activity might not qualify for a depressive episode, thus being overlooked in these patients while still increasing the risk of glucose abnormalities and diabetes.

### Is the link between ADs and diabetes due to ascertainment bias?

Some authors suggest that the association between exposure to ADs and new-onset diabetes is due to ascertainment bias. In other words, patients taking ADs would have a higher probability of a chance diagnosis of diabetes because they are more often prescribed blood examinations by their health care providers [14]. In the Whitehall II study Kivimaki and colleagues, analyzing subjects who were followed-up for more than 15 years, found that those on AD treatment at baseline were not displaying modifications in either fasting glucose or glucose levels after OGTT over time compared to the non-exposed [12]. More recently the DESIR study, following a cohort of 4700 French subjects over 9 years of follow-up, confirmed the previous results: no difference between AD users and non-users was found in either fasting glucose or insulin sensitivity [13]. However, some methodological issues make the interpretation of these studies difficult. First, differences in glucose and insulin levels at follow-up visits were evaluated after excluding patients who had already been incidentally diagnosed with diabetes outside the study. The exclusion of these diabetic subjects, presumably carrying the highest values of fasting glucose and insulin levels, is likely to cause an underestimation of the possible effect of the ADs. This is a phenomenon akin to what occurs in the analysis of time-to-event data when censoring is not correctly taken into account [52]. Second, in the DESIR study participants were considered exposed even if they reported taking ADs at just one follow-up visit, which was indeed the case for the two thirds of exposed participants: this could have caused non-differential exposure misclassification, further underestimating the association.

There is another issue that should be taken into account when evaluating the results of the aforementioned longitudinal studies. Apart from medication exposures, depression itself is a well-acknowledged risk factor for diabetes [15, 53, 54], and patients are treated with ADs most often due to underlying depression. Notwithstanding this, and although in the Whitehall II study patients on ADs were also more sedentary at baseline, both the Whitehall II and the DESIR study found that patients exposed and non-exposed to ADs displayed about the same glucose and HOMA levels change over time, implicitly stating that neither ADs nor underlying depression play any role in worsening glucose homeostasis. Those results are difficult to interpret, further suggesting that the conclusions of the studies should be taken cautiously.

According to these considerations it appears that these highly phenotyped studies could not be conclusive on downplaying the association between exposure to ADs and the risk of incident diabetes. Future longitudinal studies should not exclude patients diagnosed with diabetes by their general practitioner during the follow-up. On the other hand, information on the number of glucose tests prescribed to study subjects could be directly used in order to control for the possible role of ascertainment bias. Interestingly, the only study that did it so far found a result in line with our meta-analysis [38].

### May different ADs carry different risks of type 2 diabetes?

ADs are not equals, especially concerning side effects. Albeit the current classification may be useful to predict some of the common side effects associated with AD use, such as SSRI-induced nausea or dry mouth and constipation with tricyclics, it might be not as informative on the ability of an AD to induce diabetes. On the other hand a pharmacodynamic-based classification, built on the capacity of single ADs to interact with those receptors linked with weight gain and metabolic abnormalities, could be more useful. In a previous clinical study we showed that only exposure to ADs with high H1-receptor (H1-R) affinity, and not exposure to ADs as a whole, was associated with metabolic syndrome in patients with bipolar disorder [55]. Furthermore, in a reanalysis of the Serretti and Mandelli meta-analysis of studies assessing weight gain with ADs, we observed that the ability of ADs to induce weight gain was predicted by their H1-R affinity [56]. It is plausible to expect that the weight-gain associated with the use of high H1-R affinity ADs can eventually translate in a higher risk of developing diabetes. This hypothesis is partially supported by the study of Derjiks and colleagues that used the World Health Organization (WHO) Adverse Drug Reaction Database to evaluate the effect of ADs on glucose metabolism. The authors found that hyperglycemia was associated with the use of ADs with high affinity for H1 and 5HT2c receptors [29]. Interestingly the DPP study, the one that reported the strongest magnitude of the effect (RR: 2.41) in our meta-analysis, excluded those patients treated with either bupropion or fluoxetine, some of the ADs with the lowest affinity for H1-R [36]. It looks evident that, in order to provide more accurate and informative results, future studies should be powered to evaluate diabetes risk of single ADs, rather than lumping them together as it has been done so far.

## Conclusions

This updated meta-analysis confirms the association between AD use and incident diabetes. While it still remains a matter of debate whether this association is causal or not, in our opinion none of the biases proposed by previous authors seem able to fully explain it. It is also unclear whether single ADs exert a different effect on the risk of diabetes. Future studies should be aimed at evaluating the impact of single ADs on the incidence of diabetes; given the possible heterogeneity of effect, we suggest that a classification of ADs according to their pharmacological profiles could be useful in better elucidating the nature of this association.

## Supporting information

S1 Appendix Search strings used for the systematic review. (DOCX)

S1 PRISMA Checklist PRISMA checklist. (DOC)
