# Supplementary material for: The risk of new-onset diabetes in antidepressant users – A systematic review and meta-analysis
Source: PLoS One. 2017 Jul 31;12(7):e0182088. doi: 10.1371/journal.pone.0182088 (PMC5536271; doi:10.1371/journal.pone.0182088)
Supplement: S1 Appendix — (DOCX) [file pone.0182088.s001.docx]

**S1 Appendix.** Search strings used for the systematic review.

**PubMed search:**

**String #1.**

Filters: period 01/01/2000-31/12/2016, English language, human studies

(“Depressive Disorder/Complications”[Mesh] OR “Depression/drug therapy*”[Mesh] OR “Antidepressive Agents”[Majr] OR “Serotonin Uptake Inhibitors/therapeutic use”[Mesh] OR “Serotonin and Noradrenaline Reuptake Inhibitors/therapeutic use”[Mesh] OR “Adrenergic Uptake Inhibitors” [Mesh]) AND (“Diabetes Complications”[Mesh] OR “Diabetes Mellitus/psychology”[Mesh] OR “Diabetes Mellitus/ chemically induced“[Mesh] OR “Diabetes Mellitus/epidemiology”[Mesh] OR “Hyperglycemia”[Mesh]) AND (Risk OR Odds OR Hazard)

**String #2. The second string was used to identify relevant papers published from 2014 to 2016 that still did not have Mesh terms attached at the time of the search.**

Filters: period 01/01/2014-31/12/2016, English language, human studies.

Antidepress* AND Diabet*

**PsycINFO search:**

Filters: period 01/01/2000-31/12/2016, English language, human studies

Antidepress* AND Diabet*

**International Pharmaceutical Abstract (IPA) search:**

Filters: period 01/01/2000-31/12/2016, English language, human studies

Antidepress* AND Diabet*
